# Development of In Vitro Evaluation System for Assessing Drug Dissolution Considering Physiological Environment in Nasal Cavity

**DOI:** 10.3390/pharmaceutics14112350

**Published:** 2022-10-31

**Authors:** Daisuke Inoue, Ayari Yamashita, Hideto To

**Affiliations:** 1 Department of Medical Pharmaceutics, School of Pharmacy and Pharmaceutical Sciences, University of Toyama, 2630 Sugitani, Toyama 930-0194, Japan; 2 Molecular Pharmaceutics Laboratory, College of Pharmaceutical Sciences, Ritsumeikan University, 1-1-1 Noji-higashi, Kusatsu, Shiga 525-8577, Japan

**Keywords:** nasal formulation, powder formulation, nasal absorption, nasal mucus, Calu-3 cell line, mucosal permeation, in vitro evaluation, dissolution, 3D-printing

## Abstract

Estimating the dissolution behavior of a solid in the nasal mucus is challenging for solid dosage forms designed for the nasal application as the solid dissolves into nasal mucus and permeates through the mucosa. In the current study, the dissolution behavior of powders in the artificial nasal fluid was investigated using a 3D-printed chamber system to establish in vitro evaluation system for the dissolution of solid formulations that can simulate the intranasal environment in vivo. The dissolution rates of the five model drugs correlated with their solubility (*r*^2^ = 0.956, *p* < 0.01). The permeation rate of drugs across the Calu-3 cell layers after powder application depends on the membrane permeability of the drug. An analysis of membrane permeability considering the dissolution of powders showed the possibility of characterizing whether the drug in the powder was dissolution-limited or permeation-limited. This suggests that critical information can be obtained to understand which mechanism is more effective for the improvement of drug absorption from powders. This study indicates that the elucidation of drug dissolution behavior into nasal mucus is an important factor for the formulation of nasal powders and that the in vitro system developed could be a useful tool.

## 1. Introduction

The intranasal route for drug administration has several potential [1] delivery routes for many pharmaceutical modalities, such as small molecular compounds, peptides, proteins, nucleic acids, and antibodies [2,3,4,5]; for direct delivery of drugs to the brain region [6,7,8]; and for poorly oral absorbable drugs [9,10]. Regarding solid formulations for intranasal application, some dosage forms, such as powders [11,12,13], films [14,15,16], and lipid nanoparticles [3], were attempted in numerous investigations reported previously. The solid formulations applied intranasally dissolve into the nasal mucus containing a low water content in the nasal cavity, which then leads to drug permeation through the nasal mucosa and absorption. However, the dose of the solid formulation is limited due to the small volume of the nasal cavity, forcing the drug to dissolve in a limited amount of mucus [17,18]. The intranasal formulation is cleared toward the pharynx by nasal mucociliary clearance [19,20,21]. Prolonging the residence time in the nasal cavity or improving the dissolution property of the formulation are effective strategies. The powder dosage form is considered suitable for nasal drug delivery because of its advantages of high stability, simpler composition with the excipients, and possibility for larger dose administration compared with the liquid formulations [22]. Some studies have succeeded in improving drug dissolution and absorption with poorly water-soluble active pharmaceutical ingredients (APIs) [23], including lamotrigine [24], flurbiprofen [25], and levodopa [26]. Powder formulations are highly compatible with various pharmaceutical modalities, and the powder delivery system has been applied to nasal formulations, such as mucosal vaccines [11,27,28] and protein formulations [29,30]. In the development of solid dosage forms for nasal administration, including powder formulations, the estimation of the dissolution behavior of APIs from solid formulations into nasal mucus is essential as an alternative tool to in vivo studies, leading to a decrease in the use of animals for testing. Although ex vivo and in vitro experiments using excised animal mucosa or cultured cell lines were utilized for the permeability study, an in vitro evaluation system that can simulate the dissolution behavior of solid formulations applied to the nasal cavity considering the nasal physiological environment has not been established yet. In the current study, we developed an in vitro system to evaluate the drug dissolution of powder formulations into nasal mucus. A dissolution chamber for observing the dissolution behavior that simulated the nasal physiological environment was constructed by 3D printing, and the chamber was used for the evaluation of drug dissolution of powder formulations into an artificial nasal fluid (ANF). Furthermore, an assessment was conducted to clarify the effect of the dissolution behavior of powders on the APIs dissolution and permeation process into the mucosal membrane by comparing the dissolution behavior of APIs from powder formulations with the permeation profiles obtained from the in vitro Calu-3 permeation study.

## 2. Materials and Methods

### 2.1. Materials

Antipyrine, salicylic acid, norfloxacin, acyclovir, sulfanilic acid, methanol, acetonitrile, 0.1% trifluoroacetic acid, and mucin from porcine stomach were purchased from Fuji film Wako pure reagents (Tokyo, Japan). DMEM/F12 (Dulbecco’s modified Eagle medium: nutrient mixture F-12), fetal bovine serum (FBS), and antibiotic–antimycotic agents were purchased from Thermo Fisher Scientific (Waltham, MA, USA). Phosphate-buffered saline (PBS) and 4-(2-hydroxyethyl)-1-piperazine-ethanesulfonic acid (HEPES) were purchased from Nacalai Tesque Inc. (Kyoto, Japan). The transport medium for the membrane permeation study for Calu-3 cell layers consisted of NaCl (136.89 mM), KCl (5.36 mM), Na_2_HPO_4_ (0.34 mM), KH_2_PO_4_ (0.44 mM), MgSO_4_·7H_2_O (0.41 mM), glucose (19.45 mM), CaCl_2_ (1.26 mM), MgCl_2_·6H_2_O (0.49 mM), NaHCO_3_ (4.17 mM), and HEPES (10.00 mM).

### 2.2. Drug Dissolution from Powders to Artificial Nasal Fluid (ANF)

#### 2.2.1. Preparation of ANF

According to a previously reported study [31], ANF was prepared as an alternative to nasal mucus. Briefly, 4 *w*/*v*% mucin was dispersed in PBS, and the suspension was stirred for 24 h. After centrifugation at 16,100× *g* for 20 min twice, the obtained supernatant was used as ANF, and the pH of the ANF was adjusted to 6.4, which is equivalent to physiological nasal mucus [17].

#### 2.2.2. Preparation of Powder Dosage Forms

In order to assess the applicability of in vitro dissolution system developed, the powder dosage forms were prepared without any polymer as excipients. APIs were mixed with lactose by using an agate mortar, and the powder formulation containing 10% API was obtained. Model drugs with different physicochemical properties, including solubility and permeability, were selected for this study. The properties of these model drugs were referred from a previous report [32], and the values are listed in Table 1.

#### 2.2.3. 3D-Printing of a Dissolution Chamber

**Figure 1:**
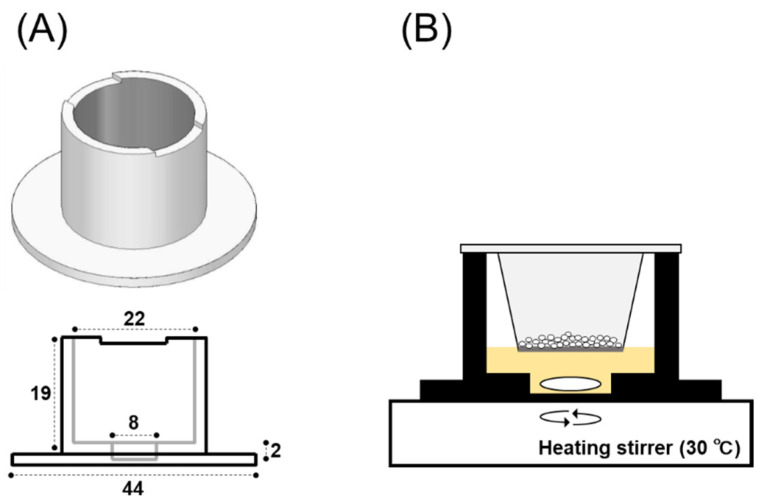
Schematic representation of the in vitro evaluation system on the dissolution of powders. (**A**) The schematic design of the dissolution chamber constructed by 3D printing. The scale is expressed in unit of mm. (**B**) The system for evaluating powder dissolution into the artificial nasal fluid (ANF). The surface area applied powders is 0.9 mm^2^.

A chamber for evaluating drug dissolution into the ANF was constructed using a 3D printer (MakerBot Replicator+, Altech Co. Ltd., Tokyo, Japan) with polylactic acid filaments. In order to mount the cell culture insert (for 12 wells with a pore size of 3.0 μm, Falcon^®^, Corning Incorporated, Corning, NY, USA) in the chamber to allow the powders to come into contact with the ANF, the dissolution chamber was designed to set the claws of the insert, and a hole was made in the bottom to accommodate the magnetic bar (2 × 8 mm) for stirring the ANF during the experiment (Figure 1A).

#### 2.2.4. In Vitro System for Evaluating Powder Dissolution into Nasal Mucus

In order to estimate the drug dissolution from powder formulations into the nasal mucus, an in vitro system was developed, considering the physiological environment of the nasal cavity. The morphometric findings in the nasal cavity and thickness of the nasal mucus layer in rats [18,33] and humans [17,34] are summarized in Table 2. By assuming practical clinical use, the volume of ANF was determined to be 0.7 mL based on the relationship between the dose of powders administered and the volume of nasal mucus present. The temperature of the ANF was kept constant at 30 °C in the dissolution chamber using a heating stirrer (Vial Hot Stirrer HSH-10VA; AS ONE Co., Osaka, Japan) to simulate the nasal mucus present in the physiological intranasal environment [35] (Figure 1B). The powders were dosed at 10 mg based on the previous studies investigated in rats [36] and humans [22]. The powder was filled into the tip for the micropipette (for 200 μL) and applied to the insert with 0.2 mL of compressed air using a syringe (1.0 mL) equipped with a three-way stopcock (Terufusion^®^; Terumo Co., Tokyo, Japan). After powder dosing, a 10 μL aliquot of ANF was collected from the bottom of the chamber at appropriate intervals of 1, 3, 5, 10, 20, 30, 45, and 60 min. In order to evaluate the dissolution behavior of the API tested in ANF, the dissolution rate constant (*k*_dis_) was determined using the initial dissolution rate, that is, the rate of change in the dissolved amount of API calculated in 1–3 min. The amount of API dissolved was calculated from the change in the concentrations of API in ANF samples.

### 2.3. Drug Dissolution and Permeation Study for Calu-3 Cell Layers

#### 2.3.1. Culture of Calu-3 Cell Layers

In accordance with a previously reported study [31], the Calu-3 cell line was cultured under air-interfaced conditions, and the Calu-3 cell layers were used for investigating in vitro powder dissolution and drug permeation study. Briefly, the Calu-3 cell line (ATCC, HTB-55, passage numbers between 21st and 32nd) was cultured in a medium of DMEM/F12 supplemented with 10% FBS and 5% antibiotic–antimycotic agent. The Calu-3 cells were seeded at a concentration of 5 × 10^5^ cells/mL on a cell culture insert and incubated at 37 °C in an atmosphere of 5% CO_2_. The Calu-3 cells were cultured under submerged conditions for 24 h after seeding, and the culture environment was changed to the air-interfaced condition by removing the media on the apical side. The integrity of Calu-3 cell layers was observed by measuring the transepithelial electrical resistance (TEER) value, and the cell membrane was used for the test after confirming that the TEER value increased and reached a constant value [31].

#### 2.3.2. Drug Permeation Study for Powder and Solutions

The permeability of the drugs across the Calu-3 cell layers was investigated by selecting five model drugs tested in the dissolution study. The experimental values of Calu-3 permeability were obtained from the previous study [31], except for salicylic acid. Briefly, the drugs were dissolved in a transport medium (pH 7.4) at a concentration of 1 mM and used as a test solution. The apical media was applied with 0.8 mL of test solution, and the basolateral media was changed to 2.0 mL. Samples (100 µL) were collected from the basolateral side over 60 min at intervals of 5, 10, 15, 20, 30, 40, 50, and 60 min. The apparent membrane permeability coefficient (*p*_app_) from the apical to the basolateral side was calculated using the following Equation (1):

*p*_app_ = δQ/δt × 1/(*A* × *C*_0_), (1)

where δQ/δt represents the rate of membrane permeability across the Calu-3 monolayers (nmol/s), *C*_0_ represents the initial concentration of the test drug on the apical side (mM), and *A* represents the surface area of the membrane (cm^2^).

### 2.4. Powder Dissolution and Drug Permeation across Calu-3 Cell Layers

#### 2.4.1. Drug Dissolution and Permeation following Powder Application

An in vitro study on drug permeation through the Calu-3 cell layers was conducted to estimate drug dissolution and permeation of the nasal mucosal membrane after the powder formulations. Calu-3 cell layers were preincubated for 15 min with 0.8 mL and 2.0 mL of transport medium on the apical and basolateral sides, respectively. In order to evaluate the effect of mucus solution on drug dissolution and permeation, the apical surface of the Calu-3 membrane was adapted with 5 μL of ANF before applying the powder on the membrane surface. Fresh transport medium (1.5 mL) was applied as the basolateral medium, and 0.1 mg of the test powder formulation was sprayed with 0.1 mL of compressed air in the device mentioned above. The actual dose of powder applied was determined from the net residual amount of powder in the tip, which is the difference in the total weight of powder in the tip before and after the application. Basolateral media (100 μL) were sampled over 120 min at intervals of 10, 20, 30, 45, 60, 90, and 120 min.

#### 2.4.2. Permeation Study for Drug Solution

The drug permeation through the cell layer after the powder dissolved was evaluated by applying a small dose of the drug solution to the Calu-3 cell layers. By applying 10 μL of 0.1 *w*/*v*% drug solution in PBS to the surface of the Calu-3 membrane, the total dose of the API was the same as that of the powder formulation (10 μg as API). After preincubation of Calu-3 cell layers for 15 min, a fresh transport medium was added to the basolateral side, and the apical surface was treated with 5 μL of ANF. The test solution (10 μL) was applied to the surface of the membrane, and 100 μL medium was sampled at intervals of 10, 20, 30, 45, 60, 90, and 120 min.

### 2.5. Sample Treatments

The mucus sample (10 μL) was deproteinated by adding nine times the volume of methanol, followed by vortexing; the supernatant (70 μL) was used for the analysis, which was obtained by centrifugation for 3 min at 16,100× *g*. The drug concentrations of the samples obtained from the drug dissolution study and in vitro Calu-3 transport study were determined by HPLC analysis (1200 Series, Agilent Technologies, Inc., Santa Clara, CA, USA). Samples were directly injected into the HPLC for the Calu-3 transport study. The HPLC analysis was conducted under an isocratic condition, and the flow rate of the mobile phase (0.1% trifluoroacetic acid/ acetonitrile = 70/30) was 1 mL/min. The temperature of the column (CAPCELL PAK, MG-II, C18, 3 μm, 4.6 mm I.D., 35 mm, Osaka Soda, Japan) was maintained at 40 °C. The volume of sample injection was 10 μL, and the retention time of APIs was 1.54, 1.28, 1.23, 0.66, and 0.51 min for antipyrine, salicylic acid, norfloxacin, acyclovir, and sulfanilic acid, respectively.

### 2.6. Statistical Analysis

The results are presented as the mean ± SEM. Statistical significance was determined using JMP^®^ software (JMP 16.2, SAS Institute Japan, Tokyo, Japan) based on a one-way analysis of variance. A *p* < 0.05 was considered to indicate the statistical significance.

## 3. Results

### 3.1. Drug Permeation Study across Calu-3 Cell Layers

In order to clarify the fundamental physicochemical properties of the APIs used, the apparent permeability coefficient (*p*_app_) through the Calu-3 layers was obtained. Since the findings on the model APIs, except for salicylic acid, were investigated in our previous report [31], we carried out the permeation study for salicylic acid in this study, and the others were referred from a previous report. The *p*_app_ values of the model APIs are listed in Table 3.

### 3.2. Powder Dissolution into ANF

**Figure 2:**
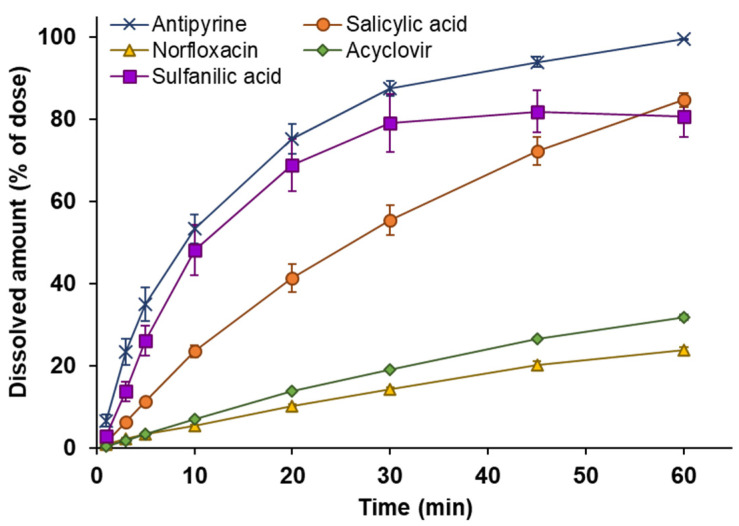
Time profiles of drug dissolution from powder formulation into ANF for five model drugs, antipyrine (×), salicylic acid (●), norfloxacin (▲), acyclovir (◆), and sulfanilic acid (■). Powder (10% API in lactose, 10 mg) was applied to the ANF. The data are represented as the mean of independent experiments, with the vertical bars indicating the S.E. (n = 3–5).

**Figure 3:**
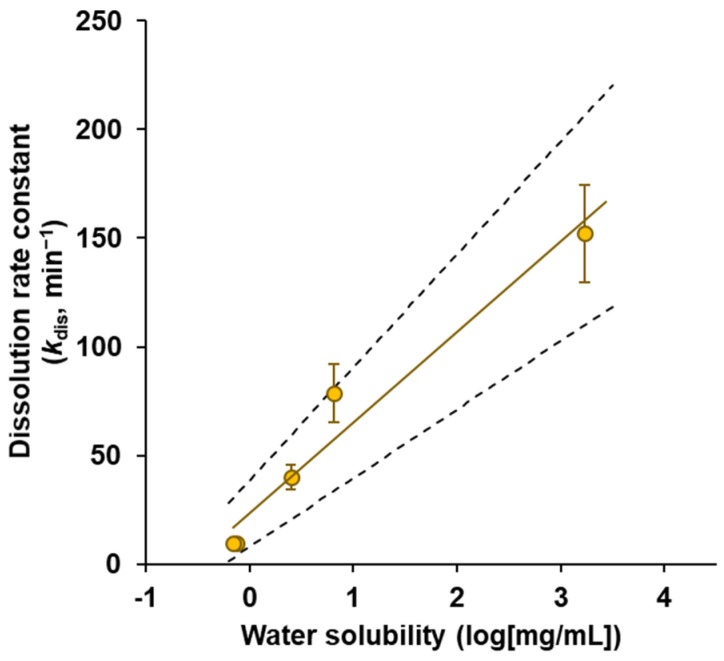
Correlation between the dissolution rate constant (*k*_dis_) and the water solubility for five model drugs, antipyrine, salicylic acid, norfloxacin, acyclovir, and sulfanilic acid. A statistically significant linear correlation was obtained (*r*^2^ = 0.956, *p* = 0.0028). The dashed lines indicate the 95% confidence interval for the fitted line. The data are represented as the mean of independent experiments, with the vertical bars indicating the S.E. (n = 3–5).

The dissolution behavior of the powders in ANF was evaluated using an in vitro system mimicking the nasal physiological environment. The time changes in the dissolved amount for powder formulations containing model APIs are shown in Figure 2. In the dissolution behavior of the powders, it was observed that the dissolution rate gradually decreased over 60 min. The dissolution rate of APIs from the powders was calculated as the change in the dissolved amount of API per unit time obtained from 1 to 3 min, which is considered to have a sufficient concentration gradient that can be regarded as a sink condition. The dissolution rates of the APIs from the powders are listed in Table 4. The dissolution rate was dependent on APIs solubility in water (Table 1). A significant linear correlation was observed between dissolution rate and water solubility (*r*^2^ = 0.956, *p* = 0.0028), as shown in Figure 3.

### 3.3. Powder Dissolution and Drug Permeation across Calu-3 Cell Layers

**Figure 4:**
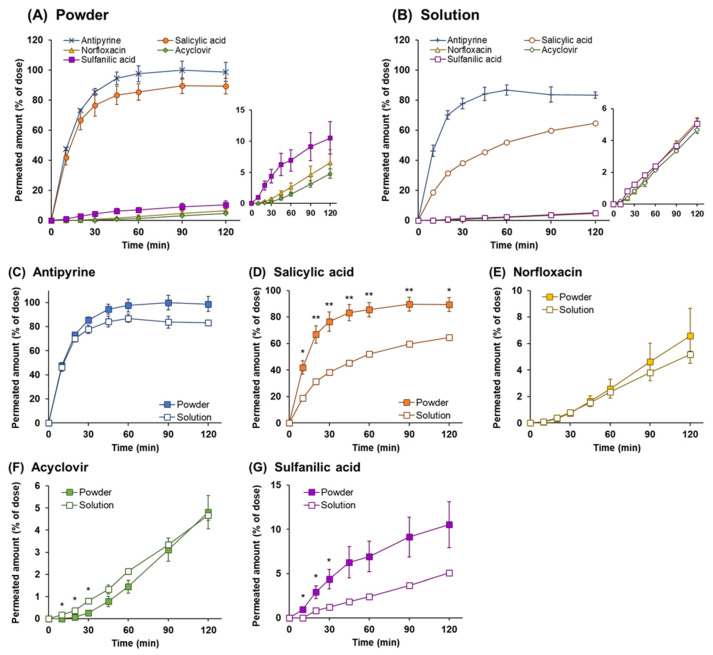
Time profiles of drug permeated amount through Calu-3 cell layers. Drug permeation behaviors are shown for the following: (**A**) powder and (**B**) solution applications using five model drugs, antipyrine (×), salicylic acid (●), norfloxacin (▲), acyclovir (◆), and sulfanilic acid (■). The profiles with different vertical axis for norfloxacin, acyclovir, and sulfanilic acid are represented in a separate panel on the right of each graph. (**C**–**G**) The characteristics between the dosage forms are shown in a graph with each model drug as a solid square for powder and an opened square for solution. The data are represented as the mean of independent experiments, with the vertical bars indicating the S.E. (n = 3–5). Statistical significances are represented in ** *p* < 0.01 and * *p* < 0.05 compared with each dosage form.

The dissolution behavior of the powders on the epithelial membrane was evaluated by investigating the powder dissolution and drug permeation through the Calu-3 cell layers. In order to elucidate the dissolution behavior in the nasal environment, a comprehensive observation of the powder dissolution and drug permeation process for powders applied on the surface of the Calu-3 layers was conducted. The time course of drug permeation through the Calu-3 layers following powder application is shown in Figure 4A. High lipophilic APIs with relatively high-water solubility, such as antipyrine and salicylic acid, rapidly permeated through the Calu-3 layers, and the permeation rates were steady within 60 min following powder application (97.6% for antipyrine and 85.5% for salicylic acid at 60 min). In contrast, the low lipophilic APIs, such as norfloxacin, acyclovir, and sulfanilic acid, had very low permeation rates regardless of their solubility, with less than 10% permeated amount at 120 min (6.6% for norfloxacin, 4.8% for acyclovir, and 10% for sulfanilic acid).

### 3.4. Drug Permeation across Calu-3 Cell Layers for Drug Solution

In order to estimate the permeation behavior of dissolved drugs through the Calu-3 cell layers, a Calu-3 permeation study with the drug solution was performed. The profiles of drug permeation from the apical side to the basolateral side after APIs application as a drug solution are shown in Figure 4B. The rapid membrane permeation was obtained for antipyrine and salicylic acid, which have high lipophilicity (Table 1). Although salicylic acid is more lipophilic than antipyrine, a lower permeation rate was obtained. Because salicylic acid has relatively high transport properties in the direction from the basolateral to the apical side [37], it was assumed that the relatively low permeation rate from the apical to basolateral side across the Calu-3 layers was observed because of the contribution of bidirectional transport [38]. For the low lipophilic APIs, i.e., norfloxacin, acyclovir, and sulfanilic acid, the membrane permeation rate was very low, and the permeated amounts at 120 min were found to be 5.2% for norfloxacin, 4.7% for acyclovir, and 5.1% for sulfanilic acid, respectively. The permeation rate of these APIs is considered to be dependent on their physicochemical properties.

### 3.5. Comparison of the Drug Permeation Behavior between Powder and Solution Dosage Forms

The effect of the dosage forms on mucosal permeation was evaluated by comparing the membrane permeation behavior after the application of the powder and the solution. The drug permeation profiles following powder and solution application are presented in Figure 4C–G. The permeation rates of API with relatively high solubility, i.e., antipyrine, salicylic acid, and sulfanilic acid, were facilitated in the powder formulation compared to that in solution, although no significant difference was observed for antipyrine. The dissolved amounts of API at 120 min in powder and solution were 98.74 % and 83.33% for antipyrine, 89.4% and 65.8% for salicylic acid, and 10.5% and 5.1% for sulfanilic acid, respectively. In contrast, a significant difference in the profiles was observed initially for acyclovir, the poorly water-soluble APIs, i.e., acyclovir showed higher membrane permeability for solution compared to powders, although a slight amount was permeated. This result can be attributed to the low solubility and low membrane permeability of acyclovir. The amount of drug dissolved on the surface of mucosa after powder application is smaller than that for a solution due to the low solubility of acyclovir, resulting in the amount permeating the membrane also being reduced. It was considered that the powder formulation for acyclovir had a dissolution rate-dependent membrane permeability. In addition, norfloxacin showed no effect on the permeation behavior in the powder and the solution forms. Since the permeated amounts were very low (4.7% for the solution at 120 min), it was assumed to have a substantially similar permeability in powder and solution form.

### 3.6. Analysis of Dissolution and Permeation of Drugs after Powder Application

**Figure 5:**
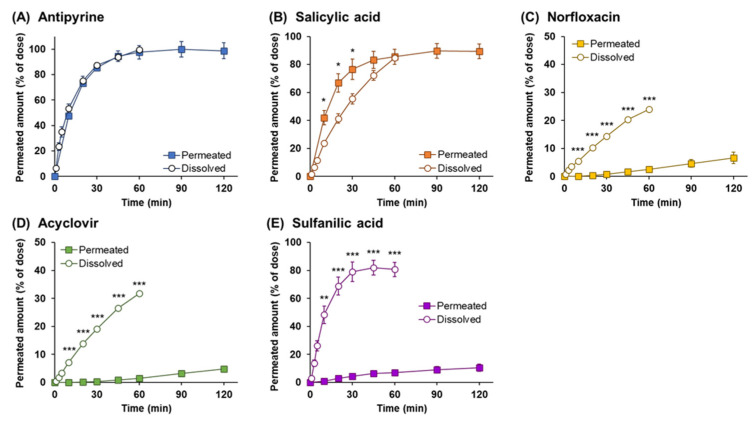
Profiles of powder dissolution and drug permeation following powder application for five model drugs: (**A**) antipyrine, (**B**) salicylic acid, (**C**) norfloxacin, (**D**) acyclovir (**E**), and sulfanilic acid. The data on the dissolution for ANF (○) were derived from the dissolution study using in vitro evaluation system (Figure 2), and the data on the powder dissolution and drug permeation (■) were derived from the Calu-3 permeation test following powder application (Figure 4A). The data are represented as the mean of independent experiments, with the vertical bars indicating the S.E. (n = 3–5). Statistical significances are represented in *** *p* < 0.001, ** *p* < 0.01, and * *p* < 0.05 compared with each condition.

The powder dissolution behavior in ANF and the drug dissolution and permeation across Calu-3 cell layers were compared to elucidate the effect of the dissolution process on the membrane permeation of the drug following powder application. The time profiles of the drug dissolved into ANF derived from the in vitro powder dissolution test and the drug permeated through Calu-3 cell layers following powder application are shown in Figure 5. Antipyrine and salicylic acid rapidly dissolved and permeated, with no time lag between powder dissolution and drug permeation, indicating that this result was due to the physicochemical properties of these APIs. Norfloxacin, acyclovir, and sulfanilic acid showed significantly slow permeation, although it can be assumed that sufficient drug is dissolved more than that permeated through the Calu-3 cell membrane. At 60 min after powder application, the amount of drug dissolved was 23.9% for norfloxacin and 31.8% for acyclovir, while the drugs permeated through Calu-3 cell layers were 2.6% and 1.4%, respectively. In the case of sulfanilic acid, although 80% of the drug was dissolved within 30 min because the solubility was relatively high, the drug permeated was significantly less than the drug dissolved, which was 6.9% at 60 min and 10.5% at 120 min, respectively.

## 4. Discussion

Drugs applied intranasally can be absorbed easily and rapidly through nasal mucosa because of the abundant capillary network under the mucous membrane and leaky epithelial transport compared with intestinal mucosa [39,40,41]. However, the volume of the nasal cavity is relatively small, and the mucosal surface area associated with drug absorption is limited compared to other routes of administration, such as the gastrointestinal tract. Therefore, many pharmaceutical strategies have attempted to achieve a sufficient therapeutic effect by enhancing the amount of absorption of drugs [42,43,44,45,46]. In particular, the solid dosage form is considered a highly effective strategy for nasal formulations because the solid formulations can increase the dose of APIs to be delivered as compared to other drug applications, such as in the solution form. Regarding the powder formulations, the dissolution rate and residence time in the nasal cavity differ depending on the preparation method, and various pharmaceutical techniques have been applied, including spray drying [19,25,47,48] and freeze drying [28,49,50] to improve the dissolution property of powders into the nasal mucus. The dissolution and diffusion behaviors of the solid and gel for the nasal formulations were estimated in an ex vivo study using excised nasal mucosa from rabbits [25,51], sheep [52], and bovine [53]. In the ex vivo experiments, the diffusion chamber, including the Franz-type diffusion cell, was used to estimate formulation dissolution and drug permeation. Although the properties of dissolution/diffusion into the fluidic media and membrane permeability through the nasal mucosa can be comprehensively evaluated for solid and microparticulate formulations, the processes of dissolution and permeation cannot be evaluated individually by ex vivo studies. Therefore, to evaluate the dissolution behavior of the solid dosage forms in the nasal cavity, we developed an in vitro evaluation system for the dissolution of solid dosage forms considering the physiological environment in the nasal cavity. With the system developed in the present study, the applied dose was 10 mg for 0.7 mL according to the physiological condition of the nasal cavity. Based on the surface area of the nasal epithelium and thickness of the mucus layer, i.e., 1343 mm^2^ and 10–20 μL in rats and 16,000 mm^2^ and 15–20 μL in humans (Table 2), the amount of mucus in the nasal cavity was calculated to be 0.027 mL in rats and 0.32 mL in human. The volume of ANF used in the system was 26 times that used in rats and 2.2 times in humans. According to the previous reports, the dose of powder applied in rats was approximately 0.5 to 1.0 mg [36,54]. In addition, regarding the marketed products for the nasal powder formulations, the single spray doses were set to 15 mg for Rhinocort^®^ powder spray and 3 mg for API for Baqsimi^®^ nasal powder. Based on these findings, the dose of the powder applied to the system was determined. Here, when powder with a dose of more than 10 mg was applied, the dissolution behavior could not be accurately evaluated because the contact area between the powder and ANF was reduced due to the limitation of the insert surface area (0.9 cm^2^).

In the current study, since system validation was needed initially for the newly developed system, small molecules were used as APIs, and a powder prepared by mixing the API and lactose, which has a simple dissolution behavior, was tested as a model of powder formulations. The dissolution behavior of the APIs was dependent on water solubility (Figure 2), and a linear correlation (*r*^2^ = 0.956, *p* > 0.01) was observed between the dissolution rate and solubility (Figure 3). Regarding the dissolution rate of the powders, the higher the solubility, the larger the dissolution rate; the order obtained was antipyrine > sulfanilic acid > salicylic acid > norfloxacin > acyclovir (Table 3). These results indicated that the in vitro system developed could be used for the quantitative evaluation of dissolution behavior in nasal mucus. In this study, the dose of powder was set corresponding to the volume of mucus in the nasal cavity for mimicking the intranasal environment. In actuality, when Calu-3 membrane permeability was assessed under similar conditions, the dissolution behavior of APIs from powder and the membrane permeation profile of the APIs showed a consistent profile (Figure 4). Therefore, although the dissolution behavior was observed under a non-sink condition in this system, it is considered not to deviate from the actual dissolution behavior in the nasal cavity. There are physiological functions that cause the drug to disappear from the nasal cavity, including the reduction in the drug dissolved in the nasal mucus due to membrane permeation and the discharge of the drug dissolved in mucus due to mucociliary clearance. Although to accurately estimate the dissolution behavior of powders, it is necessary to consider drug disappearance from mucus or the clearance and subsequent reproduction of mucus; simple evaluation may be difficult in the system incorporating these complex and various factors. The identification of important factors and the optimization of system conditions will be addressed in a future study. In actual powder manufacturing, the powders may have various characteristics, including differences in the content (number and amount of additives), powder types (particle shapes, amorphous solid dispersions, and microparticles), powder properties (particle size, size distribution, fluidity, and viscoelasticity after dissolution), and API physicochemical properties (crystallinity, solubility, and molecular weight), it is presumed that the powder formulations will have a complex dissolution process. The detailed evaluation of the powder formulations for practical use will be the next subject of interest; we will also study various solid formulations for intranasal application in the future by using the evaluation system developed in the current study.

The drugs classified in BCS classes 1, 3, and 4 were used for the evaluation. The estimation of dissolution behavior for the BCS class 2 drugs is an important challenge in order to clarify the usefulness of the system developed. The BCS class 2 drugs have a poorly water-soluble property, and the estimation considers to be difficult for the first step validation of the system because the dissolution behavior and the molecular mechanism of the dissolution process in nasal mucus are complicated. Since the primary purpose of this study was the establishment of a system for powder dissolution into the nasal cavity under a physiological condition, the model drugs should be used that have already been evaluated and have a clear correlation with in vivo parameters. Therefore, the relationship between the dissolution behavior and the water solubility was clarified as the first validation step for the newly developed system (Figure 2 and Figure 3). Although only five model drugs were evaluated in this study, the quantitative correlation obtained is a beneficial finding for developing in vitro dissolution systems for nasal formulations. We are currently proceeding with the verification of drugs with various dissolution behaviors, including BCS class 2 drugs, and increasing the number of drugs to be tested.

In order to clarify the relationship between the dissolution rate and the physicochemical properties, the initial dissolution rate constant (*k*_dis_) was calculated as an indicator to assess whether the solubility can be estimated using this system (Figure 3). The results revealed that the powder dissolution into the nasal mucus corresponding to the solubility for the APIs could be estimated by using this system, i.e., the results indicate the possibility to be a simple and rapid screening tool that can estimate the dissolution into nasal mucus for various APIs and solid formulations. In addition, the detailed mechanism of the dissolution and permeation of powders can be elucidated by considering the dissolution profile and the membrane permeation comprehensively (Figure 4). A comparative analysis between the profiles of the dissolution and Calu-3 membrane permeation suggested the possibility of accurate estimation for the dissolution and membrane permeation processes in the nasal cavity. The estimation of the complex process of powder dissolution and drug permeation into the nasal cavity can be clarified using in vitro evaluation systems.

In order to estimate drug permeation through the nasal mucosa, a permeation test was conducted using Calu-3 cell layers. Since in our previous study, it was clarified that the in vivo drug permeability through the nasal mucosa could be estimated by Calu-3 membrane permeability [31], the permeation test using Calu-3 cell layers was conducted to evaluate the drug permeation process. Based on the previous report [31], model drugs that have been known to be absorbed primarily by passive diffusion were selected and used for quantitatively estimating the mucosal permeability of the APIs.

The viability of the Calu-3 membrane after the application of the powders and solutions was confirmed by measuring the transepithelial electrical resistance (TEER) at the end of the experiment. The TEER values are listed in Table 5. Since all of the TEER values were maintained at 75% or more of the initial value, it was clarified that the powder and solution used in this study did not significantly affect the viability of the Calu-3 membrane [31].

Two different experimental systems were used to evaluate the process of membrane permeation of the dissolved drug, and both processes, including powder dissolution and drug permeation. For the former evaluation, a small volume of API solution (10 μL) was applied to the apical side of the Calu-3 membrane treated with 5 μL ANF. The drug permeation rate depended on membrane permeability (Figure 4B and Table 4), indicating that the permeation behavior of the dissolved drug can be observed using this method. Although the value of LogP was higher with salicylic acid than with antipyrine (Table 1), the membrane permeability of the Calu-3 cell layers was lower for salicylic acid. According to the results on Calu-3 permeability, the *p*_app_ was found to be 3.88 ± 0.08 for antipyrine and 1.43 ± 0.02 for salicylic acid, respectively (Table 4). Based on previous findings on the transport pathway for these two drugs [37,38], the relationship between antipyrine with high membrane permeability depending on lipophilicity and the apparent permeability with the bidirectional transportability of salicylic acid was observed to be reasonable. In addition, the permeation rate of the low-lipophilic APIs was very low, and the permeation behavior was similar to the *p*_app_ value, which was 0.081 ± 0.001 for norfloxacin, 0.067 ± 0.002 for acyclovir, and 0.044 ± 0.004 for sulfanilic acid, respectively. The relationship between the *p*_app_ and drug permeation rate obtained was consistent, indicating that the drug permeation behavior can be estimated based on the membrane permeability of the API itself by the in vitro system using a small dose of solution. In the latter evaluation, the powder was applied on the apical side of the Calu-3 membrane, and the amount of drug that permeated after powder dissolution was evaluated. The permeation rate of APIs with high lipophilicity was rapid, while that of APIs with low lipophilicity was poor for 120 min (Figure 4A). In order to estimate the effect of the physicochemical properties of APIs on the powder dissolution and drug permeation on the epithelial membrane, the permeation behaviors of APIs after the application of the powder and solution were compared (Figure 4C–G). In the powder dosage form, because a difference in permeability between the powder and solution was observed for drugs with relatively high-water solubility, it was considered that once the water on the surface of the epithelial membrane was drawn into the applied powder, the membrane permeability was partially increased by transiently inducing a high concentration gradient of the drug. Although the permeation was not significantly different in the case of antipyrine due to its high solubility and permeability, the facilitating effect of the permeability of the powders was observed in salicylic acid and sulfanilic acid. This phenomenon may be substantiated by the particularly large differences in the initial time point after powder application of salicylic acid and sulfanilic acid. In contrast, for APIs with poor water solubility, i.e., norfloxacin and acyclovir, because a transiently high concentration gradient caused by drawing water was not generated, the drug dissolved on the membrane could gradually permeate depending on the membrane permeability of the drugs.

The powder formulation following intranasal application was dissolved into nasal mucus, and the dissolved drug permeated through the nasal epithelial membrane. For powder formulations, it is essential to evaluate the processes of powder dissolution and drug permeation separately to optimize the formulation design for powders that enable precise control of drug absorption following intranasal application. Therefore, since there is no system that evaluates only the dissolution process in the nasal cavity and reproduces the physiological environment, we attempted to develop an in vitro evaluation system for powder dissolution mimicking the intranasal physiologic environment. Furthermore, in the present study, by comparing the dissolution and permeation of APIs through Calu-3 cell layers after powder application with the dissolution behavior of powders into ANF obtained by using the in vitro system developed, the effect of the dissolution process on the properties of the dissolution and membrane permeation of powders in the nasal cavity was evaluated (Figure 5). The findings obtained here suggest that it is possible to elucidate the dissolution behavior on the membrane surface and subsequent drug permeation properties by separately evaluating the processes of powder dissolution and drug permeation. Furthermore, the results suggest that powder dissolution and drug permeation in the nasal mucosa following intranasal powder application can be estimated using in vitro systems. We plan to investigate whether it is possible to estimate powder dissolution and drug permeation after in vivo application based on the data on dissolution properties obtained from the in vitro system established.

In the present study, we evaluated the dissolution behavior using an in vitro system mimicking a physiological environment in the nasal cavity. In this system, the dissolution process on the surface of nasal mucus under physiological conditions can be estimated with a similar scale to practical nasal application in vivo. In addition, because of the compact structure of this system, it has the potential to be applied to high-throughput screening of various compounds and formulations. Since in vitro evaluation systems such as the Franz cell system has been used for estimating the drug dissolution and permeation, these systems can be used for estimating the dissolution behavior of powders for nasal application. As a future project, it will be necessary to compare the system developed in this study with various systems, including the Franz cell system, and to consider the verification and optimization of a system suitable for estimating the powder dissolution process.

## 5. Conclusions

In the current study, we established an in vitro evaluation system for the dissolution of solid dosage forms following intranasal application. The results suggest that the dissolution behavior of the powders into the nasal mucus can be evaluated using an in vitro system mimicking the physiological environment in the nasal cavity, considering the scales of intranasal conditions in humans and rodents. If the dissolution of the solid formulations can be clarified in the early stages of development, it is believed that it can act as a support for the development of nasal formulations, and the promotion and expansion of formulation development for intranasal application can be anticipated.

## Figures and Tables

**Table 1:** Physicochemical properties of model drugs.

Drug	Water Solubility		LogP	BCS Class
	(mg/mL)	(log[mg/mL])		
Antipyrine	1700	3.23	0.23	1
Salicylic acid	2.51	0.40	2.26	1
Norfloxacin	0.75	−0.12	−1.03	4
Acyclovir	0.7	−0.15	−1.95	3
Sulfanilic acid	6.5	0.81	−0.9	3

**Table 2:** Morphological differences between the nasal cavity and mucus in rat and human.

			Rat	Human
Nasal cavity	Length	(mm)	91 ± 0.3 ^a^	100–140 ^c^
	Volume	(mm^3^)	256.7 ± 4.1 ^a^	20,000 ^c^
Surface area of epithelium	Squamous	(mm^2^)	44.2 ± 5.2 ^a^	-
	Respiratory	(mm^2^)	623.1 ± 14.0 ^a^	-
	Olfactory	(mm^2^)	675.2 ± 43.0 ^a^	200–400 ^c^
	Total	(mm^2^)	1343.5 ± 55.0 ^a^	16,000 ^c^
Thickness of mucus layer	Periciliary layer	(μm)	5–10 ^b^	5 ^d^
	Surface layer	(μm)	1–10 ^b^	10–15 ^d^

^a^ Data are with reference to reference [33]. The values are shown as the mean of three animals ± S.D. ^b–d^ Data are obtained from references [17,18,34].

**Table 3:** Membrane permeability across Calu-3 cell layers (*p*_app_) with five model drugs.

Drug	Calu-3 Permeability *p*_app_ (×10^−5^ cm/s)		
Antipyrine	3.88	±	0.08
Salicylic acid	1.43	±	0.02
Norfloxacin	0.081	±	0.001
Acyclovir	0.067	±	0.002
Sulfanilic acid	0.044	±	0.004

Data are presented as the mean ± S.E. of independent experiments (n = 3–6). The *p*_app_ values were obtained from our previous study [31], except for salicylic acid. A permeation test for salicylic acid was conducted in this study. Keywords: *p*_app_, apparent permeability coefficient across Calu-3 cell layers.

**Table 4:** Dissolution rate constant derived from the in vitro dissolution study using five model drugs.

Drug	Dissolution Rate Constant *k*_dis_ (min^−1^)		
Antipyrine	152.12	±	22.53
Salicylic acid	40.10	±	5.59
Norfloxacin	9.89	±	1.62
Acyclovir	9.76	±	0.29
Sulfanilic acid	78.84	±	13.37

Data are presented as the mean ± S.E. of independent experiments (n = 3–5). Keys: *k*_dis_, dissolution rate constant calculated from the dissolved amounts of powder at initial time points from 1 to 3 min.

**Table 5:** Transepithelial electrical resistance (TEER) values of Calu-3 cell layers following drug application as solution and powder dosage forms.

Drug	TEER (% of Initial)					
	Solution			Powder		
Antipyrine	91.04	±	1.63	94.82	±	1.12
Salicylic acid	82.24	±	3.78	75.30	±	2.76
Norfloxacin	88.32	±	1.95	84.37	±	3.71
Acyclovir	83.35	±	1.81	94.98	±	1.80
Sulfanilic acid	93.87	±	7.72	84.74	±	2.46

Data are presented as the mean ± S.E. of independent experiments (n = 3–5).

## Data Availability

Not applicable.

## Abbreviations

ANF, artificial nasal fluid; API, active pharmaceutical ingredient; *k*_dis_, dissolution rate constant; *P*_app_, apparent membrane permeability coefficient; TEER, transepithelial electrical resistance.
